# Supplementary material for: ProteinShader: illustrative rendering of macromolecules
Source: BMC Struct Biol. 2009 Mar 30;9:19. doi: 10.1186/1472-6807-9-19 (PMC2672931; doi:10.1186/1472-6807-9-19)
Supplement: Additional file 1 — ProteinShader program without source code. This compressed file contains the complete ProteinShader program including associated libraries, but no source code. A README.txt file gives an overview of the ProteinShader distribution, and the index.html file in the help subdirectory has directions on getting started with the program as well as a set of tutorials. [file 1472-6807-9-19-S1.zip › ProteinShader-beta-0_9_4-binary/help/api/org/proteinshader/graphics/typography/TextLabelManager.html]

TextLabelManager (ProteinShader API)


|  |  |  |
| --- | --- | --- |
| **Overview** \| **Package** \| **Class** \| **Use** \| **Tree** \| **Deprecated** \| **Index** \| **Help** | |  |
| **PREV CLASS**   NEXT CLASS | **FRAMES**    **NO FRAMES**     **All Classes** |  |
| SUMMARY: NESTED \| FIELD \| CONSTR \| METHOD | DETAIL: FIELD \| CONSTR \| METHOD |  |


---


## org.proteinshader.graphics.typography Class TextLabelManager

```
java.lang.Object
  org.proteinshader.graphics.typography.TextLabelManager
```

---

``` public class TextLabelManager extends Object ```

Knows how to create and cache OpenGL display lists holding the
glTexSubImage() with a bitmap for a text label that can be pasted
onto curved surfaces.

---

| **Field Summary** | |
| --- | --- |
| `static int` | `BYTES_PER_PIXEL`             The blank texture map has 2 bytes per pixel. |
| `static String` | `MAP`             The 'textlabel.frag' fragment shader uniform variable for a 2D texture map is named 'textLabelMap'. |
| `static int` | `TABLA_RASA_HEIGHT`             The blank texture map is 128 pixels high. |
| `static int` | `TABLA_RASA_WIDTH`             The blank texture map is 128 pixels wide. |
| `static int` | `X_OFFSET`             The x-offset for pasting a subimage is 47 pixels. |
| `static int` | `Y_OFFSET`             The y-offset is 0 pasting a submimage is 0 pixels. |


**Constructor Summary**

`TextLabelManager()`
  
          Constructs a TextLabelManager.


| **Method Summary** | |
| --- | --- |
| `void` | `cacheDisplayLists(GL gl, List<String> textLabels)`             Caches an OpenGL display list holding glTexSubImage2D( bitmap ) for each textLabel in the list given as an argument. |
| `void` | `deleteDisplayLists(GL gl)`             Frees graphics card memory for any OpenGL display lists currently being used to store text labels. |
| `void` | `deleteTablaRasa(GL gl)`             Frees graphics card memory for the tabla rasa. |
| `void` | `enableTexture(GL gl, String segmentID, int shaders, RegionEnum regionType)`             Enables OpenGL texture mapping and binds a texture with text cooresponding to the segmentID given as an argument. |
| `void` | `loadTablaRasa(GL gl)`             Loads a blank texture map, the reusable tabla rasa. |
| `void` | `readGlyphSet()`             Obtains a set of glyphs by using the default '.conf' configuration file in the fonts directory. |
| `void` | `setDebug(boolean debug)`             Setting debug to true will turn on debugging (which prints data to standard out). |

**Methods inherited from class java.lang.Object**

`clone, equals, finalize, getClass, hashCode, notify, notifyAll, toString, wait, wait, wait`

**Field Detail**

### MAP

```
public static final String MAP
```

:   The 'textlabel.frag' fragment shader uniform variable for a 2D
    texture map is named 'textLabelMap'.

    **See Also:**: Constant Field Values

---


### X\_OFFSET

```
public static final int X_OFFSET
```

:   The x-offset for pasting a subimage is 47 pixels.

    **See Also:**: Constant Field Values

---


### Y\_OFFSET

```
public static final int Y_OFFSET
```

:   The y-offset is 0 pasting a submimage is 0 pixels.

    **See Also:**: Constant Field Values

---


### TABLA\_RASA\_WIDTH

```
public static final int TABLA_RASA_WIDTH
```

:   The blank texture map is 128 pixels wide.

    **See Also:**: Constant Field Values

---


### TABLA\_RASA\_HEIGHT

```
public static final int TABLA_RASA_HEIGHT
```

:   The blank texture map is 128 pixels high.

    **See Also:**: Constant Field Values

---


### BYTES\_PER\_PIXEL

```
public static final int BYTES_PER_PIXEL
```

:   The blank texture map has 2 bytes per pixel.

    **See Also:**: Constant Field Values


**Constructor Detail**

### TextLabelManager

```
public TextLabelManager()
```

:   Constructs a TextLabelManager.


**Method Detail**

### cacheDisplayLists

```
public void cacheDisplayLists(GL gl,
                              List<String> textLabels)
```

:   Caches an OpenGL display list holding glTexSubImage2D( bitmap )
    for each textLabel in the list given as an argument.

---


### deleteDisplayLists

```
public void deleteDisplayLists(GL gl)
```

:   Frees graphics card memory for any OpenGL display lists currently
    being used to store text labels.

    :   **Parameters:**: `gl` - the current GL object.

---


### enableTexture

```
public void enableTexture(GL gl,
                          String segmentID,
                          int shaders,
                          RegionEnum regionType)
```

:   Enables OpenGL texture mapping and binds a texture with text
    cooresponding to the segmentID given as an argument.

---


### loadTablaRasa

```
public void loadTablaRasa(GL gl)
```

:   Loads a blank texture map, the reusable tabla rasa.

---


### deleteTablaRasa

```
public void deleteTablaRasa(GL gl)
```

:   Frees graphics card memory for the tabla rasa.

    :   **Parameters:**: `gl` - the current GL object.

---


### readGlyphSet

```
public void readGlyphSet()
                  throws GlyphException
```

:   Obtains a set of glyphs by using the default '.conf' configuration
    file in the fonts directory.

    :   **Throws:**: `GlyphException` - if a problem occurs while trying to read the set of glyphs from a file.

---


### setDebug

```
public void setDebug(boolean debug)
```

:   Setting debug to true will turn on debugging (which prints data
    to standard out).

    :   **Parameters:**: `debug` - boolean value to turn debugging on or off.


---


|  |  |  |
| --- | --- | --- |
| **Overview** \| **Package** \| **Class** \| **Use** \| **Tree** \| **Deprecated** \| **Index** \| **Help** | |  |
| **PREV CLASS**   NEXT CLASS | **FRAMES**    **NO FRAMES**     **All Classes** |  |
| SUMMARY: NESTED \| FIELD \| CONSTR \| METHOD | DETAIL: FIELD \| CONSTR \| METHOD |  |


---

# *Copyright © 2007-2008*
